# Associations of long chain polyunsaturated fatty acids with bone mineral density and bone turnover in postmenopausal women

**DOI:** 10.1007/s00394-022-02933-9

**Published:** 2022-07-30

**Authors:** Orlagh Feehan, Pamela Jane Magee, Laura Kirsty Pourshahidi, David John Armstrong, Mary Martina Slevin, Philip James Allsopp, Marie Catherine Conway, J J Strain, Emeir Mary McSorley

**Affiliations:** 1 Nutrition Innovation Centre for Food and Health (NICHE), Ulster University, Coleraine, BT52 1SA UK; 2 Department of Rheumatology, Altnagelvin Hospital, Western Health and Social Care Trust, Londonderry, BT47 6SB UK

**Keywords:** LCPUFA, Bone mineral density, Postmenopausal women, Bone resorption

## Abstract

**Purpose:**

The immunomodulatory properties of n-3 long chain polyunsaturated fatty acids (LCPUFA) are reported to reduce bone loss through alteration of bone remodelling and n-3 LCPUFA, therefore, may benefit bone health in post-menopausal women, a vulnerable group at high risk of osteoporosis.

**Methods:**

Measures of bone mineral density (BMD) were determined using dual energy X-ray absorptiometry (DEXA) in 300 post-menopausal women. The bone turnover markers osteocalcin (OC), C-terminal telopeptides of type 1 collagen (CTX) and total alkaline phosphatase were quantified in serum along with urinary creatinine corrected deoxypyridinoline (DPD/Cr) and CTX/Cr and the CTX:OC ratio calculated. Total serum n-6 PUFA (LA + AA) and n − 3 LCPUFA (ALA + EPA + DPA + DHA) were measured and the n − 6:n − 3 ratio was calculated.

**Results:**

Mean (SD) age and body mass index (BMI) were 61 (6.4) years and 27.4 (4.8) kg/m^2^, respectively with participants being 12.6 (7.6) years post-menopause. Multiple regression analysis identified no association between n-3 LCPUFA and any of the measures of T-score or BMD albeit a significant positive association between total n − 3 LCPUFA and femur BMD (*β* = 0.287; *p* = 0.043) was observed within those women with a low n − 6:n − 3 ratio. There was a significant inverse association between ALA and urinary DPD/Cr (*β* = − 0.141; *p* = 0.016).

**Conclusion:**

A favourable low n − 6:n − 3 ratio was associated with higher femur BMD and a higher n − 3 LCPUFA (ALA) was associated with lower bone resorption. These results support a beneficial role for n − 3 LCPUFA in reducing postmenopausal bone resorption and favourably influencing BMD.

**Trial number & date of registration:**

ISRCTN63118444, 2nd October 2009, “Retrospectively registered”.

## Introduction

Osteoporosis is a chronic debilitating condition characterised by a decrease in bone mineral density (BMD) and altered bone microarchitecture, which can lead to structural fragility and a subsequent increased risk of fracture [1, 2]. Osteoporosis affects 3 million people in the United Kingdom (UK) and annually over 8.9 million osteoporotic fractures occur worldwide [3, 4]. Postmenopausal women are at increased risk of developing osteoporosis and associated fractures as a result of the natural decline of oestrogen after the menopause [5]. Declining oestrogen concentrations increase bone resorption by enhancing osteoclastogenesis and also reduce new bone formation activity by osteoblasts, causing a net loss of bone tissue [6]. Greater expression of pro-inflammatory cytokines as seen after menopause and in later age is one possible contributing factor in the development of osteoporosis. Hormones (such as oestrogen), pro- and anti-inflammatory cytokines play an important role in regulating osteoblast and osteoclast differentiation and activity. A balance of these systems is needed to prevent the development of osteoporosis [7, 8]. It has been postulated that n − 3 LCPUFA can up-regulate the absorption of calcium from the intestine and reduce the production of prostaglandin E2 (PGE2) which is a key eicosanoid involved in the modulation of inflammation [9]. Elevated concentrations of PGE_2_ can lead to a reduction in the production of osteoprotegerin (OPG), an important osteoclastogenesis inhibitory factor expressed by osteoblasts. This production in turn can increase the expression of receptor activator of nuclear factor kappa-Β ligand (RANKL) as a result of decreased OPG [10] leading to overall negative effects on bone health.

Nutrition is a modifiable factor important in optimising bone health [11] with well-defined roles for protein, calcium and vitamin D in reducing the risk of osteoporosis and fragility fractures post menopause [12]. Recent evidence suggests that n − 3 long chain polyunsaturated fatty acids (LCPUFA) may benefit bone health and prevent bone loss [13–16]; however, there is limited evidence in postmenopausal women. Furthermore, the majority of research to date has focused on dietary intakes of n − 3 LCPUFA rather than the biological status of n − 3 LCPUFA in postmenopausal women. Dietary n − 3 LCPUFA have been proposed to benefit overall bone health [15] and have a role in reducing the risk of hip fracture in all age groups [17]. A higher intake of total n − 3 LCPUFA and fish have been positively associated with BMD [17–19] and a reduced risk of hip fracture [20] as well as total fracture risk in postmenopausal women [11, 21]. Positive associations between n − 3 LCPUFA status, dietary n − 3 LCPUFA and skeletal outcome measures such as lumbar spine and femur BMD have been demonstrated in older people and in postmenopausal women and are postulated to be owing to higher fish consumption [13, 22]. Total dietary n − 6 PUFA and the n − 6:n − 3 ratio have been shown to be negatively associated with spine and femur BMD in cohorts of older adults [13, 23] and postmenopausal women [24] albeit studies were limited by use of a nutrient database or dietary records to quantify dietary n − 3 LCPUFA intake rather than status [23, 24]. Relative amounts of n − 6 and n − 3 LCPUFA should be taken into consideration for overall health, with research suggesting a lower n − 6:n − 3 ratio could be of greater benefit to bone health [23].

Recent analysis of the UK biobank including 492,713 adults showed a significant association between habitual fish oil supplement use and lower risk of both incident and recurrent fractures [25]. A limited number of supplementation studies with n − 3 LCPUFA have been conducted in postmenopausal women with the majority reporting an inverse association between n − 3 LCPUFA and markers of bone resorption including C-terminal telopeptides of type 1 collagen (CTX) and urinary pyridinoline (Pyd), and others reporting beneficial effects of n − 3 supplementation on the bone formation markers bone-specific alkaline phosphatase (BALP) and osteocalcin (OC) in postmenopausal women [2, 26]. Although the reported changes were small in magnitude, they warrant further investigation particularly given that inclusion of rich sources of n − 3 LCPUFA, such as oily fish, in the diet could be a simple effective dietary alteration in these women [26]. To date, the evidence is limited by small sample sizes and short study durations and research is needed to further investigate this association between n − 3 LCPUFA and BMD in post-menopausal women [27].  Therefore, the aim of this study was to investigate the relationship between serum n − 3 LCPUFA status with BMD and BTMs in a large cohort of postmenopausal women.

## Materials and methods

### Study design

A convenient sample of a total of 300 non-osteoporotic postmenopausal women (45–75 years) were recruited to take part in a study conducted in Ulster University to investigate the effect of a combination of Aquamin™ and Nutraflora® on bone health [28]. The current study utilises baseline data from this trial. Women were excluded if they were pre- or peri-menopausal, went through menopause earlier than 40 years old, used medication or supplements known to affect bone health, had osteoporosis or a medical condition known to affect bone health. Dietary intake of total daily fish (g/day) and lifestyle factors were determined by a 4-day semi-quantitative food diary and food frequency questionnaires. Food composition database Weighed Intake Software Package (WISP) (WISP for WINDOWS, version 3; Tinuviel Software, Anglesey, United Kingdom) was used to analyse the dietary intakes. Information on total physical activity was assessed by means of a shortened version of the International Physical Activity Questionnaire [29] and was represented as MET (metabolic equivalent tasks) hours per week. Participants also completed a questionnaire about past/current medical history. Ethical approval was received from Ulster University Research Ethics Committee (REC/08/0083) and the original study was registered as a clinical trial (www.controlled-trials.com as ISRCTN63118444). The study was conducted in accordance with the declaration of Helsinki and written informed consent was obtained from all participants.

### Measurements

A non-fasted blood sample was collected from each participant. Duplicate weight (kg) and height (cm) were taken to calculate body mass index (BMI) (weight (kg)/height (m)^2^). Lumbar spine and femur BMD (g/cm^2^) and T-scores were determined using dual energy X-ray absorptiometry (DEXA) (Lunar corporation, Madison, WI). BMD was recorded from the total hip or the femoral neck depending on which was the lowest value. According to the cut-offs established by the World Health organisation [30], osteoporosis is defined as BMD at least 2.5 SD below that of a healthy young adult (T-score is < − 2.5), osteopenia when the T-score is between -1.0 and -2.5 and a normal bone density value above − 1.0. BMD was expressed to 3 decimal places and T-scores to 1 decimal place according to the International Society for Clinical Densitometry [31]. Diagnosis was made according to the site with the lowest T-score.

### Sample analysis

#### Blood and urinary bone turnover markers

Blood samples were collected from all participants for analysis. Serum bone formation marker osteocalcin (OC) (normal reference value: 26.5 (12.8–55.0 95% CI) ng/ml) and bone resorption marker C-terminal telopeptides of type 1 collagen (CTX) (normal reference value: 0.439 (0.142–1.351 95% CI) ng/ml) were measured using enzyme-linked immunosorbent assay (ELISA) (Immunodiagnostics Systems Ltd (IDS); intra-assay CV was 1.8% & 4.7%; respectively). Serum total alkaline phosphatase was quantified by Roche Modular Analyser and expressed as a bone formation marker (u/L). Urinary bone resorption markers CTX (normal reference range: 324 (121–874 95% CI) µg/mmol/Cr) and deoxypyridinoline (DPD) (7.56 (2.27)/7.94 (3.25) nmol DPD/mmol Cr) were measured using enzyme immunosorbent assay (EIA) (Urine CrossLaps EIA, IDS, intra-assay CV was 4.7% and Urine DPD EIA, Metra DPD EIA kit; Quidel, intra-assay CV was 4.3%). Urinary CTX and DPD results were both corrected for urinary concentrations of creatinine (Cr). CTX:OC ratio was calculated. A high CTX:OC ratio means higher bone resorption relative to bone formation.

#### Polyunsaturated fatty acid (LCPUFA) analysis

Serum LCPUFA were quantified using an adaption of the method by Folch et al. [32]. In brief, fatty acid methyl esters were detected and quantified by gas chromatography–mass spectrometry (7890A-5975C; Agilent) using heptadecanoic acid (C17:0) as the internal standard [33]. All analytic standards were of ≥ 99% purity and purchased from Sigma-Aldrich. Serum LCPUFA status was chosen as a biomarker to encompass recent LCPUFA concentrations of the triacylglycerol fraction. The individual n − 6 PUFAs; linoleic acid; 18:2 n − 6 (LA), arachidonic acid; 20:4 n − 6 (AA) and the individual n − 3 LCPUFA, α-linolenic acid; 18:3 n − 3 (ALA), eicosapentaenoic acid; 20:5 n − 3 (EPA), docosapentaenoic acid; 23:5 n − 3 (DPA) and docosahexaenoic acid; 22:6 n − 3 (DHA) were quantified. Results were presented as milligrams per millilitre (mg/mL). Total n − 6:n − 3 ratio was calculated. A high n − 6:n − 3 ratio is considered less favourable as this means higher total n − 6 PUFA relative to n − 3 LCPUFA.

### Statistical methods

All data analysis was performed using Statistical Package for the Social Sciences (SPSS, IBM SPSS Statistics version 25). Nominal data were presented as mean (SD) and categorical variables presented as frequencies (*n*) and percentages (%). Data were tested for normality using Kolmogorov–Smirnov statistic test and variables were transformed using the natural logarithm where appropriate. Pearson correlation analysis was conducted to test for correlation sample size.

Primary analysis assessing the associations between the natural logarithm of LCPUFA (total n − 3, n − 6, n − 6:n − 3 ratio) with femur and lumbar spine BMD and T-score was conducted using Spearman correlation analysis. Separate multiple regression analysis was also performed between femur and lumbar spine BMD and T-score as a dependent variable and LCPUFA additionally adjusting for age, BMI, years’ post-menopause, total physical activity and smoking status as independent variables. Secondary analysis was conducted to test for associations between the natural logarithm of LCPUFA (total n − 3, n − 6, n − 6:n − 3 ratio) and BTMs (OC, CTX, DPD/Cr, CTX/Cr, CTX:OC ratio) using the same statistical approach. Tertiles were created and participants were classified as having a high (≥ 6.5), medium (between 4.9 and 6.5) or low (≤ 4.9) n − 6:n − 3 ratio. Participants who were classified as having a low, medium or high n − 6:n − 3 ratio were selected as individual groups and regression analysis was repeated to take into consideration relative amounts of n − 6 and n − 3 LCPUFA and to determine if n − 6 or n − 3 LCPUFA was associated with bone parameters and BTMs, after adjusting for independent variables. To better satisfy regression assumptions, we used the natural logarithmic transformation of all markers in the models. As some of the T-scores were negative values, we added a constant of + 3 to each individual T-score data prior to natural logarithmic transformation. All values presented in tables are log transformed. Significance was set at *p* < 0.05.

## Results

### Baseline characteristics

Table 1 summarises the characteristics for the 300 post-menopausal women. Data for BTMs and PUFA concentrations were available for 299 postmenopausal women. Mean (SD) age and BMI of subjects were 61 (6.4) years and 27.4 (4.8) kg/m^2^, respectively. Mean values for years post-menopause and physical activity were 12.6 (7.6) years and 25.4 (24.3) MET hours/week. Age of menarche and menopause were 13.1 (1.5) and 48.5 (5.2) years. Mean (SD) total dietary intake of fish was 30.2 (31.8) g/day. Mean (SD) dietary intake of polyunsaturated fat was 11.43 (4.57) g/day.

**Table 1:** Descriptive characteristics of postmenopausal women (*n* = 300)

Descriptive	Mean (SD)
Age (years)	61.0 (6.4)
Height (cm)	160.6 (6.2)
Weight (kg)	70.7 (12.8)
Body mass index (kg/m^2^)	27.4 (4.8)
Years post-menopause	12.6 (7.6)
Total physical activity (MET hours/week)	25.4 (24.3)
Femur BMD (g/cm^2^)	0.895 (0.1)
Lumbar spine BMD (g/cm^2^)	1.099 (0.1)
Femur T-score	− 0.7 (0.9)
Lumbar spine T-score	− 0.6 (1.2)
*n* Normal based on T-score (%)	127 (42.3)
*n* Osteopenia based on T-score (%)	173 (57.7)
*BTMs*	*n* = 299
Serum CTX (ng/mL)	0.63 (0.20)
Serum total alkaline Phosphatase (u/L)	77.3 (19.5)
Serum Osteocalcin (ng/mL)	19.68 (0.22)
Urinary CTX/Cr (ug/mmoL Cr)	309.2 (146.6)
Urinary DPD/Cr (nmol DPD/mmol Cr)	8.1 (2.3)
CTX:Osteocalcin ratio	3.4 (1.1)
*LCPUFA*	
Total n − 3 (mg/mL)	0.241 (0.110)
Total n − 6 (mg/mL)	1.328 (0.375)
LA (C18) (mg/mL)	1.014 (0.304)
AA (C20) (mg/mL)	0.313 (0.099)
ALA (C18) (mg/mL)	0.030 (0.017)
EPA (C20) (mg/mL)	0.071 (0.064)
DPA (C23) (mg/mL)	0.037 (0.015)
DHA (C22) (mg/mL)	0.103 (0.045)
n − 6:n − 3 ratio	6.4 (3.3)

*SD* standard deviation, *MET* metabolic equivalent of task, *BMD* bone mineral density, *BTMs* bone turnover markers, *serum CTX* serum C-terminal telopeptides of type 1 collagen, *urinary CTX/Cr* urinary C-terminal telopeptides of type 1 collagen/creatinine, *urinary DPD/Cr* urinary deoxypyridinoline/creatinine, *LCPUFA* long chain polyunsaturated fatty acids, *total n3* total omega 3, *total n6* total omega 6, *LA* linoleic acid (18:2 n − 6); AA, arachidonic acid (20:4 n − 6); ALA, α-linolenic acid (18:3 n − 3); EPA, eicosapentaenoic acid (20:5 n − 3); DPA, docosapentaenoic acid (23:5 n − 3); DHA, docosahexaenoic acid (22:6 n − 3); n − 6:n − 3 ratio, total n − 3:total n − 6 ratio

### Correlation analysis

A significant negative correlation was observed between the bone resorption marker urinary DPD/Cr and total n − 3 LCPUFA (*r* = − 0.137; *p* = 0.018) as well as with EPA (r = -0.150; *p* = 0.009) and DPA (r = -0.118; *p* = 0.042). A significant negative correlation was observed between the bone resorption marker urinary CTX/Cr and AA (r = -0.121; *p* = 0.036) and ALA (r = -0.123; *p* = 0.033). There were no significant correlations observed between any measure of BMD or T-score with n − 6 or n − 3 LCPUFA. There was no significant correlation between any of the bone density parameters and the n − 6:n − 3 ratio.

### Covariates

Age was a significant negative predictor of femur (β = -0.221; *p* = 0.012) and lumbar spine BMD (β = -0.208; *p* = 0.019) but not a significant predictor of BTMs. BMI was a significant positive predictor of femur (β = 0.261; P < 0.001) and lumbar spine BMD (β = 0.327; P < 0.001). BMI was also a significant negative predictor of serum CTX (β = -0.324; P < 0.001), serum osteocalcin (β = -0.249; P < 0.001) and urinary CTX/Cr (β = -0.396; P < 0.001). The number of years’ post-menopause was a significant negative predictor of serum total alkaline phosphatase (β = -0.194; *p* = 0.033) and total physical activity was a significant negative predictor of urinary DPD/Cr (β = -0.124; *p* = 0.044) but neither were significant predictors of bone parameters. Smoking status was a significant negative predictor of femur T-score (β = -0.494; *p* = 0.002) but was not a significant predictor of BTMs.

### Relationships between bone parameters and LCPUFA

**Fig. 1:**
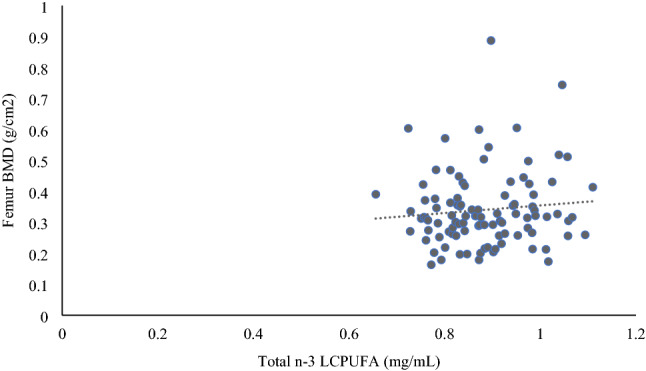
Significant positive association between total n − 3 LCPUFA and femur BMD within those with a low n − 6:n − 3 ratio (*n* = 100). Significance determined using Multiple regression adjusted for age, BMI, physical activity, smoking status, years’ post-menopause and n − 6 PUFA (*β* = 0.287, *R*^2^ with covariates only = 0.122, total model *R*^2^ with LCPUFA = 0.162, *p* = 0.043)

Associations between LCPUFA and bone parameters are outlined in Table 2. After adjusting for age, BMI, physical activity, smoking status and years’ post-menopause, the n − 3 LCPUFA, ALA, EPA, DPA, DHA and n − 6 PUFA, AA, LA were not significantly associated with the bone parameters femur and lumbar spine BMD or with T-score (P > 0.05). There was also no significant association observed between the n − 6:n − 3 ratio and bone parameters after adjusting for covariates. When split into tertiles according to the n − 6:n − 3 ratio, a significant positive association between total n − 3 LCPUFA and femur BMD (β = 0.287; *p* = 0.043) within those with a low n − 6:n − 3 ratio was observed (Fig. 1).

**Table 2:** Associations between LCPUFA and femur and lumbar spine bone mineral density and T-score in postmenopausal women (*n* = 299)

	Femur BMD (g/cm^2^)			Lumbar spine BMD (g/cm^2^)			Femur T-score			Lumbar spine T-score		
Multivariate model	*R*^2^	Std *β*	*p* value	*R*^2^	Std *β*	*p* value	*R*^2^	Std *β*	*p* value	*R*^2^	Std *β*	*p* value
Total n − 3 LCPUFA (mg/mL)	0.140	0.018	0.751	0.126	0.030	0.599	0.149	0.023	0.686	0.127	0.022	0.700
ALA (mg/mL) (C18)	0.142	− 0.047	0.399	0.125	-0.013	0.815	0.151	− 0.049	0.379	0.127	− 0.028	0.618
EPA (mg/mL) (C20)	0.140	0.015	0.784	0.126	0.033	0.561	0.149	0.017	0.754	0.126	0.017	0.762
DPA (mg/mL) (C23)	0.141	− 0.033	0.549	0.125	− 0.007	0.899	0.149	-0.020	0.724	0.126	-0.015	0.792
DHA (mg/mL) (C22)	0.142	0.042	0.462	0.127	0.050	0.386	0.150	0.039	0.493	0.127	0.035	0.542
Total n − 6 PUFA (mg/mL)	0.146	0.075	0.175	0.126	0.033	0.556	0.153	0.065	0.240	0.129	0.051	0.366
LA (mg/mL) (C18)	0.145	0.068	0.221	0.126	0.019	0.733	0.151	0.049	0.375	0.127	0.033	0.550
AA (mg/mL) (C20)	0.146	0.076	0.177	0.129	0.066	0.245	0.157	0.094	0.093	0.134	0.088	0.119
n − 6: n − 3 ratio	0.141	0.031	0.577	0.125	− 0.008	0.886	0.149	0.020	0.723	0.126	0.011	0.843

*Std β* Standardised Coefficients Beta, *BMD* bone mineral density, *LCPUFA* long chain polyunsaturated fatty acids, *total n3* total omega 3, *total n6* total omega 6, *LA* linoleic acid (18:2 n − 6), *AA* arachidonic acid (20:4 n − 6), *ALA* α-linolenic acid (18:3 n − 3), *EPA*, eicosapentaenoic acid (20:5 n − 3), *DPA* docosapentaenoic acid (23:5 n − 3), *DHA* docosahexaenoic acid (22:6 n − 3), n − 6: n − 3 ratio, total n − 3:total n − 6 ratio

^*^Denotes significance (*p* < 0.05). Multivariate model adjusted for age, BMI, physical activity, smoking status and years’ post-menopause

### Relationships between BTMs and LCPUFA

Associations between BTMs and serum LCPUFA concentrations are summarised in Table 3. After adjusting for covariates, there were no significant associations observed between total n − 3 LCPUFA, EPA, DPA or DHA with any of the markers of bone turnover or the CTX:OC ratio. There was a significant inverse association between the n − 3 LCPUFA ALA and urinary DPD/Cr (β = -0.141; *p* = 0.016). Each unit increase in ALA was associated with a -0.141 mg/mL decrease in urinary DPD/Cr. The n − 6: n − 3 ratio was not significantly associated with any BTMs. Furthermore, there was no significant association between total n − 6 PUFA, AA and LA and any of the markers of bone turnover. When split into tertiles for the n − 6: n − 3 ratio, a significant negative association between total n − 3 LCPUFA and osteocalcin (β = -0.767; *p* = 0.036) within those with a medium n − 6: n − 3 ratio was observed. There was a significant positive association between total n − 3 LCPUFA and urinary CTX/Cr (β = 0.224; *p* = 0.024) within those with a high n − 6: n − 3 ratio.

**Table 3:** Associations between LCPUFA and bone turnover markers in postmenopausal women (*n* = 299)

	Serum CTX (ng/mL)			Serum OC (ng/mL)			Serum alkaline phosphatase (u/L)			Urinary CTX/CR (ug/mmol/L)			Urinary DPD/CR (nmol DPD/mmol Cr)			CTX:OC ratio		
Multivariate model	R^2^	Std β	P value	R^2^	Std β	P value	R^2^	Std β	P value	R^2^	Std β	P value	R^2^	Std β	P value	R^2^	Std β	P value
Total n − 3 PUFA (mg/mL)	0.098	− 0.010	0.865	0.061	0.022	0.705	0.004	− 0.034	0.565	0.169	− 0.031	0.57	0.061	− 0.104	0.078	0.014	− 0.039	0.511
ALA (mg/mL) (C18)	0.100	− 0.056	0.332	0.061	− 0.015	0.792	0.036	− 0.021	0.721	0.169	− 0.040	0.469	0.070	− 0.141	0.016*	0.015	− 0.047	0.429
EPA (mg/mL) (C20)	0.098	− 0.008	0.893	0.062	0.036	0.535	0.035	− 0.003	0.966	0.172	0.069	0.209	0.062	− 0.105	0.072	0.015	− 0.055	0.362
DPA (mg/mL) (C23)	0.098	− 0.022	0.699	0.061	0.020	0.732	0.036	− 0.025	0.670	0.168	− 0.007	0.896	0.059	− 0.088	0.131	0.015	− 0.052	0.389
DHA (mg/mL) (C22)	0.098	− 0.012	0.831	0.062	0.031	0.603	0.042	− 0.081	0.174	0.168	− 0.03	0.591	0.055	− 0.062	0.297	0.015	− 0.054	0.378
Total n − 6 PUFA (mg/mL)	0.098	− 0.016	0.775	0.065	− 0.063	0.275	0.037	− 0.039	0.503	0.174	− 0.080	0.141	0.051	− 0.004	0.942	0.016	0.06	0.315
LA (mg/mL) (C18)	0.098	0.003	0.710	0.066	− 0.071	0.222	0.037	− 0.037	0.528	0.174	− 0.082	0.131	0.051	− 0.006	0.916	0.016	0.063	0.287
AA (mg/mL) (C20)	0.098	0.003	0.963	0.061	− 0.024	0.688	0.037	− 0.037	0.537	0.171	− 0.059	0.285	0.051	0.001	0.983	0.013	0.033	0.586
n − 6: n − 3 ratio	0.098	− 0.001	0.988	0.065	− 0.063	0.280	0.036	0.008	0.888	0.168	− 0.021	0.702	0.061	0.101	0.088	0.018	0.078	0.193

*Std β* Standardised Coefficients Beta, *serum CTX* C-terminal telopeptides of type 1 collagen, *serum OC* serum osteocalcin, *urinary CTX/Cr* urinary C-terminal telopeptides of type 1 collagen/creatinine, urinary DPD/Cr, urinary deoxypyridinoline/creatinine, *CTX:OC ratio, serum* C-terminal telopeptides of type 1 collagen:serum osteocalcin, *LCPUFA* long chain polyunsaturated fatty acids, *total n3* total omega 3, *total n6* total omega 6, *LA* linoleic acid (18:2 n − 6); *AA* arachidonic acid (20:4 n − 6), *ALA* α-linolenic acid (18:3 n − 3), *EPA* eicosapentaenoic acid (20:5 n − 3), *DPA* docosapentaenoic acid (23:5 *n* − 3), *DHA* docosahexaenoic acid (22:6 n − 3); n − 6:n − 3 ratio, total n − 3:total n − 6 ratio

^*^Denotes significance (*p* < 0.05). Multivariate model adjusted for age, BMI, physical activity, smoking status and years’ post-menopause

## Discussion

This observational study showed no significant association between n − 3 LCPUFA and any of the measured bone parameters in this cohort of postmenopausal women; however total n − 3 LCPUFA was shown to be positively associated with femur BMD in those postmenopausal women with a low n − 6: n − 3 ratio. Consideration should also be given to reducing n − 6: n − 3 ratio through improving dietary intake of n − 3 LCPUFA along with reducing n − 6 PUFA. In terms of BTMs, a higher ALA concentration (n − 3 LCPUFA) was significantly associated with lower urinary DPD/Cr, suggesting lower bone resorption. Overall, this study highlights that increasing n − 3 LCPUFA may have benefits to bone health in postmenopausal women, a vulnerable group at high risk of developing osteoporosis and associated fractures.

A number of studies have investigated associations between total PUFA status or n − 3 LCPUFA dietary intake and BMD in women [18, 19, 23, 34, 35] as well as in postmenopausal women [21, 22, 24, 36]. A significant positive association between dietary n − 3 LCPUFA and lumbar spine and femoral neck BMD has previously been reported [19, 22], but only after adjusting for covariates [22] including age, BMI, duration of menopausal state, grip strength, and intakes of calcium, vitamin D, vitamin K, n − 6 PUFA, polyunsaturated fatty acid, serum N-terminal propeptide of type I collagen, and urinary type-I collagen cross-linked N-telopeptide. These studies assessed dietary intake of n − 3 LCPUFA rather than serum n − 3 LCPUFA concentrations and therefore were not able to verify the correlation between n − 3 LCPUFA and BMD using a validated biomarker of status [19, 22]. In a recent study in 301 Spanish postmenopausal women, a significant positive association between plasma n − 3 LCPUFA and spine and femur neck BMD was observed after adjusting for covariates [21]. It is important to note that the Spanish cohort included osteoporotic women whereas anyone with osteoporosis was excluded from this study making it more challenging to determine associations with BMD. Nevertheless, taken together, this research supports a beneficial role n − 3 LCPUFA for bone health in postmenopausal women.

It has been postulated that higher intakes of n − 6 PUFA results in raised pro-inflammatory cytokine production that stimulates osteoclastic activity and thereby negatively impacting on bone health [37, 38]. Additionally, PGE2 produced from arachidonic acid (n − 6 LCPUFA), is the main prostaglandin involved with the bone turnover cycle with lower PGE2 promoting bone formation whereas higher concentrations of PGE2 have been shown to inhibit bone formation [18, 39]. The optimal n − 6: n − 3 PUFA ratio for favourable effects on bone health is currently unknown; however, a contemporary western diet high in n − 6 PUFA and therefore a higher n − 6: n − 3 ratio, may not contain the optimum amount of n − 3 LCPUFA needed to benefit bone health [40]. It has been hypothesised that a balance between n − 6 and n − 3 LCPUFA is of greater importance for resultant health benefits than either class of PUFA by itself [41]. This hypothesis has been supported in our study where in participants with a low n − 6: n − 3 ratio, a significant positive association between n − 3 LCPUFA and higher femur BMD was observed. Overall, circulating n − 6: n − 3 ratio was approximately 6:1 in the present study and this might suggest women in our cohort had a favourable dietary intake of n − 3 LCPUFA as the circulating ratio is relatively low when compared to a western diet ratio of around 15–20:1 [42, 43]. Previous studies in similar groups of older women have reported a range of dietary n − 6: n − 3 ratios, between 4:1 to 8:3 [21, 22, 24, 34]. In the current study, dietary intake of fish was 30.2 g/day which is equivalent to 211.4 g/week. Dietary intake of rich n − 3 LCPUFA sources such as oily fish can be low, with the current average adult intake in the United Kingdom ~ 56 g/week which is 2.5 times lower than the current guidance of at least one portion of oily fish/week (140 g) [44]. Although fish intakes in our study were shown to be sufficiently high to meet the UK recommendation, data were not available on the types of fish consumed and therefore it was not possible to quantify oily fish consumption. Fish intakes may have been primarily from non-oily fish sources. Furthermore, the inclusion of fish in the diet provides a significant source of protein and other nutrients with known benefits for musculoskeletal health and although this research was focused on PUFA it is plausible that other factors may be adding to the benefits seen. It has been reported that greater intakes of dietary n − 3 LCPUFA or use of supplementation may be essential for resultant benefits to bone health [24]. Dietary recommendations for fish consumption are based on the nutritional benefits of consuming fish especially as a rich source of n − 3 LCPUFA but also take into consideration the risks from potential pollutants [45]. In addition, there are no recommendations for n − 3 supplements [46] and therefore it is difficult to recommend an optimal n − 3 LCPUFA supplementation dose for benefit to bone heath.

BMD and BTMs have been assessed as outcome variables in several studies to investigate relationships between n − 3 LCPUFA and bone health. BTMs change more rapidly than BMD and therefore BTMs can be used to observe response to treatment before changes in BMD occur [47]. Although n − 3 LCPUFA was not significantly associated with bone formation markers, ALA (n − 3 LCPUFA) was shown to be significantly associated with less bone resorption in the present study. Similar findings have previously been reported in a group of elderly patients in which no significant association between n − 3 LCPUFA on the bone formation marker BALP but an inverse association with the bone resorption marker TRAP-5b [13]. Although intervention with a low dose (1.2 g) of n − 3 LCPUFA or fish oil showed no significant effect on bone formation markers [26, 48], intervention with 900 mg n − 3 LCPUFA for 6 months led to a significant decrease in the bone resorption marker urinary Pyd when compared to a control group in postmenopausal women [26]. In addition, intervention with much higher doses of EPA + DHA (4 g) has been reported to significantly reduce deoxypyridinoline (DPD) from baseline to 6 months when compared to the control in postmenopausal women [27] suggesting a potential beneficial role for n − 3 LCPUFA in reducing bone resorption in postmenopausal women. In those with a medium n − 6: n − 3 ratio, we observed a significant negative association between total n − 3 LCPUFA and bone formation marker osteocalcin. In addition, in those with a high n − 6: n − 3 ratio, we observed a significant positive association between total n − 3 LCPUFA and bone resorption marker urinary CTX/Cr which is contrary to what others have reported [49, 50].

We observed a significant negative association between the n − 3 LCPUFA ALA concentrations and urinary DPD/Cr. It has been postulated that n − 3 LCPUFA may lessen bone resorption by decreasing urinary excretion of calcium and reducing inflammatory cytokine production which are significant stimuli for osteoclastic activity [51, 52]. ALA may affect bone resorption through PGE2 which in turn can prevent activation of RANKL, a key growth factor that stimulates and promotes osteoclast (bone-resorbing cells) formation [53]. The cause and effect to explain the reported relationship between n − 3 LCPUFA and bone health has not been conclusively determined but it has been proposed that the immunomodulatory effects of the PUFAs may contribute to the inhibition of osteoclast formation and in turn have a beneficial effect on preventing bone loss. Advancing age and menopause are associated with greater pro-inflammatory cytokine expression and chronic inflammation, in addition to other mechanisms which can accelerate bone loss and contribute to the development of osteoporosis [54, 55]. Pro-inflammatory cytokines play a significant role in oestrogen depletion-associated bone loss in postmenopausal women [50, 56]. The LCPUFA have an immunomodulatory effect through the regulation of pro-inflammatory cytokines IL-1β, IL-6, IL-8 and TNF-α through the production of eicosanoids including prostaglandins (PGs), thromboxanes (TXs), leukotrienes (LTs) and resolvins [26, 57]. Therefore, enhancing LCPUFA in the diet, particularly n − 3 LCPUFAs such as EPA and DHA, could have resultant benefits to the inflammatory milieu [57] and favourable effects on bone remodelling in postmenopausal women [50].

Research assessing the beneficial effect of LCPUFA on bone has frequently used dietary recall methods such as a food frequency questionnaires and food diaries to determine the dietary intake of LCPUFA. These dietary instruments can lead to variations in results owing to the methodological confinements of dietary recall [58]. In this study, PUFA were quantified in serum using the gold standard method of gas chromatography–mass spectrometry; however other measures such as plasma, phospholipids and red blood cells could be alternative, cost effective and readily available methods for PUFA analysis [13]. The cross-sectional nature of this study negates the ability to determine causality. Based on Pearson’s correlation coefficient analysis, this study had over 90% power to detect correlation coefficients larger in magnitude than 0.20. Due to the challenging nature of interpreting n − 6: n − 3 ratio, secondary analysis in this study should be interpretated with caution. Measuring EPA and DHA within the red cell membrane for determination of the n − 3 index may be useful in fully elucidating the benefits of n − 3 PUFA on bone health without the confounding effect of the more pro-inflammatory n − 6 PUFA. In addition, it is possible we have residual confounding owing to lack of inclusion of additional confounding predictors of bone health e.g. vitamin D, calcium & protein. Future studies with larger sample sizes should take this into consideration. Our analysis could also have benefited from including a sub-group of postmenopausal women with osteoporosis to further explore associations between serum PUFA and osteoporotic postmenopausal women, albeit this study still adds to the body of biological research exploring the beneficial role of dietary PUFA on bone in postmenopausal women.

In conclusion, the positive association between a low n − 6: n − 3 ratio and femur BMD alongside the negative association between ALA with urinary DPD/Cr highlights a potentially important role for n − 3 LCPUFA status on maintenance of bone health in postmenopausal women. Our findings warrant further investigation using long-term intervention studies to confirm this possible beneficial role for dietary intake of n − 3 LCPUFA in reducing postmenopausal bone loss which could provide an evidence base to promote the intake of n − 3 LCPUFA rich dietary sources to support bone health.

## Data Availability

The authors confirm that the data supporting the findings of this study are available in this article.
